# First biometrics record of bartail flathead, *Platycephalus indicus* (Linnaeus, 1758) from the Bay of Bengal, Bangladesh

**DOI:** 10.1016/j.heliyon.2022.e11124

**Published:** 2022-10-18

**Authors:** Md. Rahamat Ullah, Md Mahamudul Hasan Mredul

**Affiliations:** a Bangladesh Fisheries Research Institute, Riverine Sub-Station, Khepupara, Patuakhali 8650, Bangladesh; b Faculty of Fisheries, Patuakhali Science and Technology University, Dumki, Patuakhali 8602, Bangladesh

**Keywords:** Length-weight relationships, Length-length relationships, Condition factor, Form factor, Sex ratio

## Abstract

For the very first time the sex ratio, length-weight relationships (LWRs), length-length relationships (LLRs), form factor, as well as condition factor were calculated for bartail flathead, *Platycephalus indicus*, captured with gill nets (mesh size: 2.0–6.0 cm) from the Bay of Bengal, Bangladesh, from August 2021 to January 2022. A digital caliper was used to measure the length to 0.1 cm accuracy, and an electronic balance was used to quantify weight to the accuracy of 0.01 g. The sex ratio for the sample was 1: 0.76 (Male: Female). The estimates of slope in the fitted linear regressions relating logarithms of weights to logarithms of the total, standard, and fork lengths varied from 2.978 to 3.297, and the coefficient of determination from 0.89 to 0.97. Moreover, the three-length measures were also strongly associated (r^2^ > 0.996; *P* < 0.05). For the males, females, and combined sexes, the computed form factors were 0.0111, 0.0112, and 0.0107, correspondingly. For both males and females, individuals in the 33–36 cm and 27–30 cm length classes exhibited the highest and lowest in all the four condition factors, respectively. The size at first sexual maturity for combined sex of *P. indicus* was 30.2 cm in total length in the Bay of Bengal, Bangladesh. These results of the study will help with the conservation and long-term management of this species in the Bay of Bengal, Bangladesh, and other nearby nations.

## 1 Introduction

*Platycephalus indicus* (Linnaeus, 1758), commonly known as bartail flathead, a member of the family Platycephalidae (Flatheads) and the order Scorpaeniformes, is native to the Indian Ocean (Imamura, 2015) but found in the Indo-West Pacific, Red Sea, and East Africa, as well as in the Philippines, northern Japan, and Australia (Talwar and Jhingran, 1991; Riede, 2004; Froese and Pauly, 2007). Their bodies are lengthy, with flattened heads, long tail, as well as their large mouths, with the bottom jaw considerably larger than the top (Kuiter and Tonozuka, 2001; Nelson, 2006). This sedimentary fish may be encountered on the continental shelf’s muddy or sandy grounds, at depths of less than 100 m and up to 300 m (Imamura, 2015; Froese and Pauly, 2007). It is employed by conventional healers. It possesses high energy (310 kJ/100 g), protein (18.8 g/100 g), low-fat content (0.3 g/100 g), high mineral composition (Iron, Zinc, and Calcium content 1.7 mg, 0.79 mg, and 150 mg, respectively) and has economic value (Bogard et al., 2015; Chen et al., 2020). However, there is relatively little knowledge about the physiology of this species in the Bay of Bengal, Bangladesh.

A fish’s weight is related to its length however, knowledge of these parameters would aid the estimation of productivity. The length-weight relationships (LWRs) are also widely acknowledged as the most effective framework for evaluating fish stocks inside the environment (Andrade and Camos, 2002; Saha et al., 2019, 2021), and it is of considerable relevance in species population assessments (Oscoz et al., 2005; Ogunola and Onada, 2017). The research on LWRs has grown popular, and fish scientists are now doing it on a regular basis (Froese, 2006; Saha et al., 2019; Rahman et al., 2020). Fish longevity is typically assessed by determining the ages of fish sampled from the population and growth is typically assessed by analysis of the lengths of fish at their ages at capture, tracking the progression over time of modes in length compositions, or analysis of data from recaptured fish that were tagged and released (Ogunola and Onada, 2017; Huang et al., 2018). LWRs assessment is helpful for estimating the condition of fish or plumpness score, which is an influential factor for assessing the wellbeing of fish stocks or individuals (Muchlisin et al., 2017; Batubara et al., 2019; Rahman et al., 2020), and for comparing individual life history of a species across regions (Bagenal and Tesch, 1978; Muchlisin and Siti-Azizah, 2009; Oluwatoyin et al., 2013).

Fish condition factors (K) may be used as an indicator of a fish’s overall well-being and condition in regard to its surroundings, reflecting how strong or fairly deep-bodied the fish are (Froese, 2006) and physiological status (Rahman et al., 2020; Parvin et al., 2021). This value is derived from the relationship between the length and weight of a fish in order to ascertain the condition of that organism (Nash et al., 2006; Bolarinwa, 2017). The ratio of females and males in a stock is an important phenotypic metric as well as a good indication of stock characteristics and fertility, as well as a basic principle in biological evolution (Clutton-Brock, 2007). Changes in the environment that affect the sex ratio of fish that recruit to the population, mortality that is related to sex, differential sexual habits, development rate, and lifespan expectations might all contribute to a sex ratio imbalance (Badcock and Merrett, 1976; Clarke, 1983).

Some studies (Hashemi and Taghavimotlagh, 2013; Mousavi-Sabet et al., 2015; Kassem et al., 2021) have demonstrated the morphometric traits and biology of *P. indicus* from the Persian Gulf coastal waters in Iran and Bardawil lagoon in Egypt. However, there are no published data available with accurate biological status such as sex ratio, fish size distribution, length-weight dynamics, form factor (a_3.0_) as well as condition factor (K) of *P. indicus* from Bangladeshi waters to the best of our knowledge. The aims of the present study, therefore, are to collect and report, for the first time, fundamental data on the sex ratio, fish size distribution, length-weight relationships (LWRs), length-length relationships (LLRs), form factor (a_3.0_), condition factor, size at first sexual maturity as well as natural mortality of *P. indicus* from the Bay of Bengal, Bangladesh.

## 2 Materials and methods

**Figure 1:**
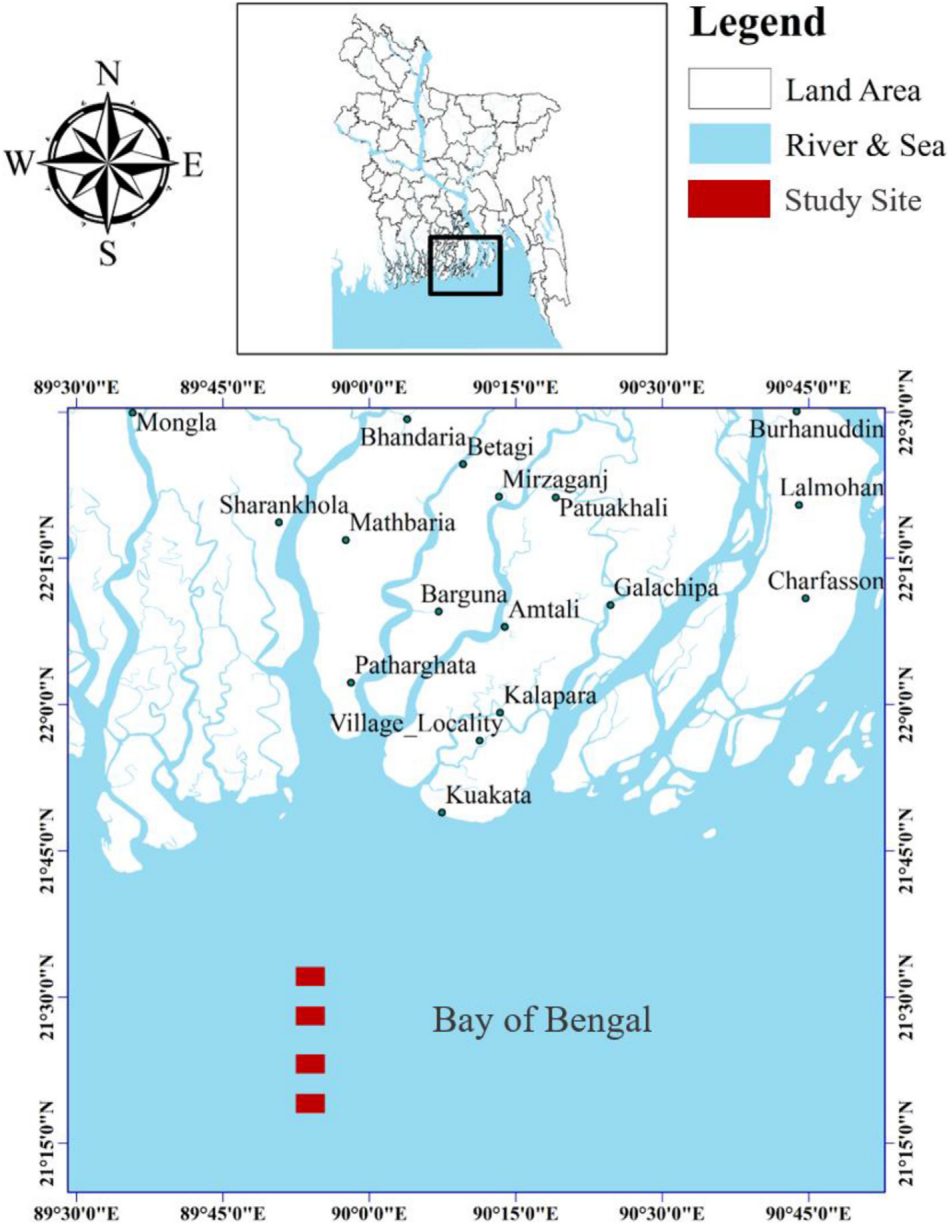
Sampling locations of bartail flathead, *Platycephalus indicus* across the Bay of Bengal, Bangladesh.

From August 2021 to January 2022, the research was carried out in the Bay of Bengal, on Bangladesh’s mid-southern coast (Figure 1). This sampling location belongs to a major fishing zone of the Bay of Bengal named ‘swatch of no ground’. Local fishermen captured fish employing gill nets (mesh size: 2.0–6.0 cm), and 197 individuals of *P. indicus* were pooled over these five months. Following Cheng and Zheng (1987), all collected fish were instantaneously placed on ice and brought to the laboratory for verification and measurement. Individuals of *P. indicus* were identified using the characteristics that distinguished different species of *Platycephalus* from Chinese seas, as described by Chen et al. (2020). The gonads of all individuals were visually inspected and sexed (Mohammadikia et al., 2014). A digital caliper was used to measure each specimen’s total length (TL), standard length (SL), and fork length (FL) were measured and recorded in cm to the nearest 0.1 cm and weight (W) was measured in g using an electronic balance and recorded to the nearest 0.01 g. The research was conducted in accordance with the ethical standards of the Bangladesh Fisheries Research Institute. The experimental protocol and guidelines were maintained according to the animal welfare and ethical committee of Bangladesh Fisheries Research Institute, Bangladesh. The research and use of animals for the experiment have been authorized by the ethical committee.

The total lengths of *P. indicus* were classified into 2 cm intervals with the total number of fish of each sex in the different intervals providing the length distribution for that sex. The relationship between weight and length of fish was assumed to be a power function of the form described by Le Cren (1951):

W = aL^b^

where L was the standard length (cm), fork length (cm), or total length (cm), and W was the total weight (g).

Estimates of the intercept (a) and slope (b), were obtained by fitting LWRs using the method described by Froese (2006) method of linear regression following logarithmic conversion, log (W) = log (a) + b log (L) where a and b represent coefficient related to body form and growth type respectively, with Length (L) and Weight (W). Just before linear regression investigation, anomalies were removed using log-log plots of length and weight (Froese, 2006). For parameters, a and b, the coefficient of determination (r^2^) and 95% confidence limits (95% CL) were also determined. Furthermore, linear regressions were employed to estimate the parameters and coefficients of determination of the fitted linear relationships between TL vs SL, TL vs FL, as well as SL vs FL. Form factor (a_3.0_) was calculated by the formula: a_3.0_ = 10^log a-s(b−3)^ (Froese, 2006). As recommended by Froese (2006), an average slope of S = −1.358 was used to estimate the form factor for the analyzed species using weight and total length. Fulton’s condition factor (K_F_) was calculated by the formula of Fulton (1904): K_F_ = 100 × (W/L^3^). The allometric condition factor (K_A_) was calculated by the equation of Tesch (1971): K_A_ = W/L^b^. The relative condition factor (K_R_) was calculated following the equation of Le Cren (1951): K_R_ = W/(aL^b^); where W and L represent the weight of the body (g) and total length (cm) respectively, a and b are the LWR parameters. The relative weight (W_R_) was calculated with the formula (Froese, 2006): W_R_ = (W/W_s_) × 100; where W is the weight of a particular individual, W_s_ is the predicted standard weight for the same individual, W_s_ is calculated by W_s_ = aL^b^. The size at first sexual maturity (L_m_) of *P. indicus* was measured through the empirical formula proposed by Binohlan and Froese (2009): log (L_m_) = ˗0.1189 + 0.9157 × log (L_max_); where L_m_ is the size at first sexual maturity in total length, L_max_ is the maximum observed total length of *P. indicus* in the present study. The Natural mortality (M_w_) of *P. indicus* was assessed through the model proposed by Peterson and Wroblewski (1984): M_w_ = 1.92 year^−1^ × (W) ^(−0.25)^; where M_w_ is natural mortality at mass weight; and W = aL^b^, a and b are the regression variables of LWR. Statistical software R (R Core Team, 2020) and GraphPad Prism 9 were used to conduct all analyses. The Spearman rank correlation test was applied to evaluate the association of condition factors with total length and body weight.

## 3 Results

### 3.1 Fish size distribution and sex ratio

**Figure 2:**
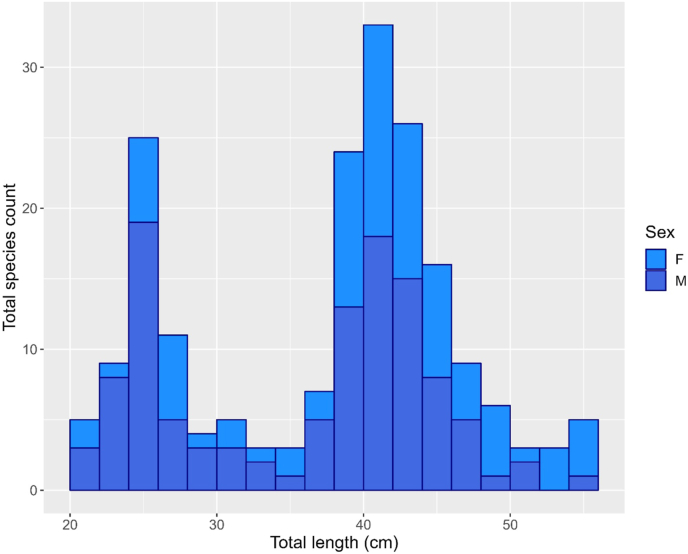
Length size distribution of bartail flathead, *Platycephalus indicus* across the Bay of Bengal, Bangladesh, X-axis represent the total length (TL) with 2 cm bar interval, Y-axis represents the number of total species count, two-color for two sexes respectively.

There were 112 males (57%) and 85 females (43%) among the 197 sampled fish, the data for which were subjected to morphometric analysis. According to the sex ratio for the entire sample was 1: 0.76 (M: F), males were much more abundant than females throughout all TL categories between 20 and 40 cm, while females are dominating from 40 to 56 cm (Figure 2). The greatest numbers of males and females were reported in the 38–40 cm (Male = 22, Female = 09) and 40–42 cm size groups (Male = 19, Female = 24), respectively.

### 3.2 Length-weight relationships (LWRs)

**Figure 3:**
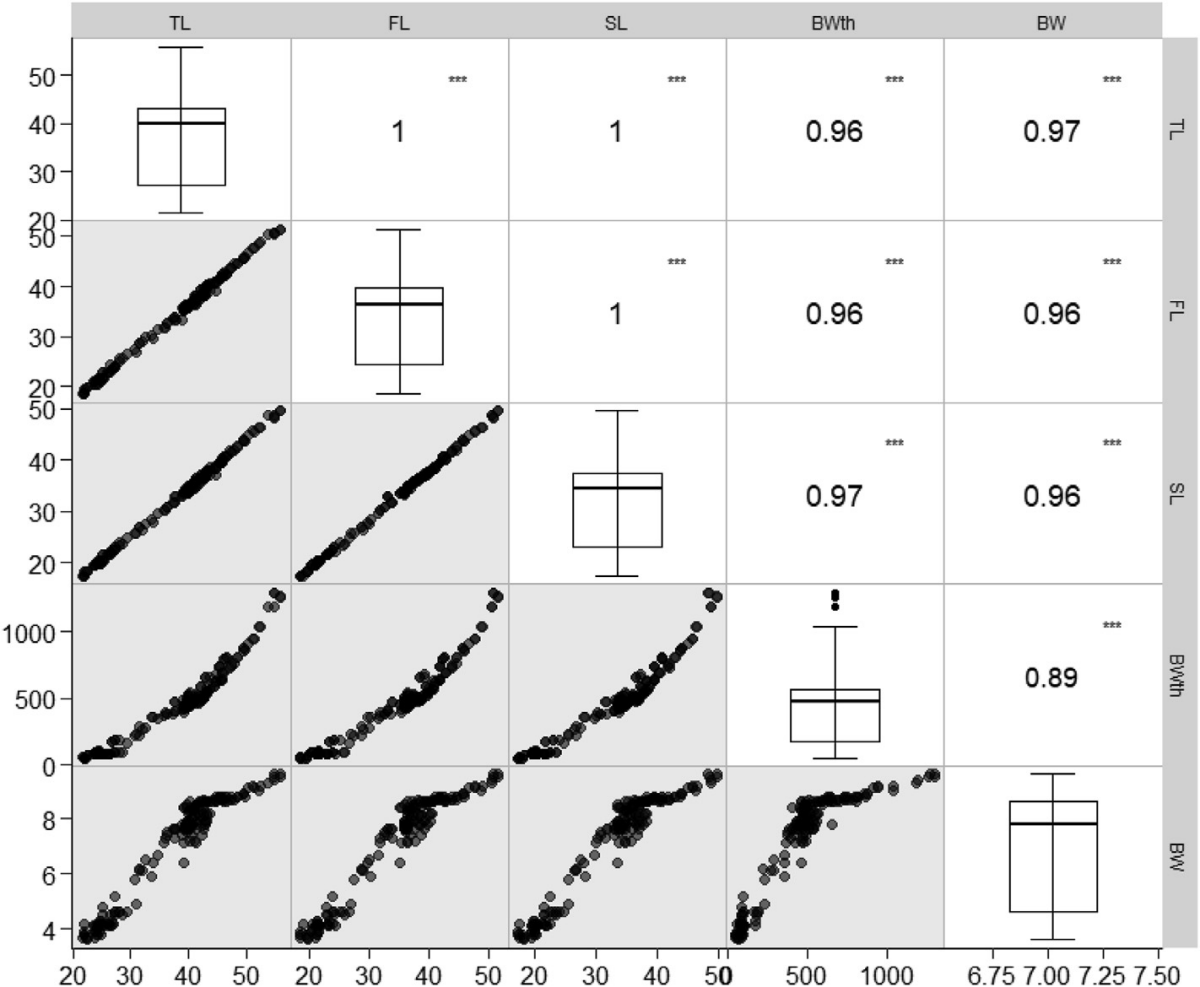
Scatter plot represents the relationship between weight and length parameters of bartail flathead, *Platycephalus indicus* across the Bay of Bengal, Bangladesh; Boxes represent a 25th to 75th percentile, solid lines in boxes are median value, error bar represent 5th and 95th percentile of individual parameters; Regression coefficient (r^2^) values are coded as number for each relationship and ∗∗∗ are constituted for a significant relationship at 0.05 level of significance.

A descriptive analysis of length-weight assessment is depicted in Table 1. A high correlation of determination value was observed between weight and length variables ranging from 0.89 to 0.97 (Figure 3). The “b” value was between 2.978 and 3.297.

**Table 1:** Length-weight relationships parameters and form factor of bartail flathead, *Platycephalus indices* from the Bay of Bengal, Bangladesh.

Sex	n	a	95% Cl of a	b	95% Cl of b	GP	Length type	a_3.0_
Male	112	0.011	0.008–0.015	3.004	2.912–3.094	I	SL	0.0111
		0.002	0.001–0.003	3.295	3.200–3.389	A+	TL	
		0.008	0.005–0.011	3.043	2.947–3.139	I	FL	
Female	85	0.012	0.008–0.017	2.978	2.864–3.091	A-	SL	0.0112
		0.002	0.001–0.003	3.296	3.173–3.419	A+	TL	
		0.007	0.005–0.011	3.054	2.935–3.173	I	FL	
Combined	197	0.011	0.008–0.014	2.994	2.925–3.062	A-	SL	0.0107
		0.002	0.001–0.003	3.297	3.224–3.369	A+	TL	
		0.008	0.006–0.010	3.047	2.975–3.119	I	FL	

Here, n: sample size, a: intercept, b: slope, TL: Total Length, BW: Body Weight, FL: Fork Length, SL: Standard Length, GP: Growth Pattern, A+: Positive Allometric, A-: Negative Allometric, I: isometric.

### 3.3 Length-length relationships (LLRs)

The estimated LLRs for *P. indicus* in the coastal region of Bangladesh showed that b values were less than 1 in male, female, and combined sex in the relationship between SL and FL but higher than 1 in all the other relationships for male, female, and combined sex (Table 2). With r^2^ values more than 0.996 and b values varying from 0.960 to 1.075, all LLRs were found statistically significant (*P* < 0.05). ANOVA shows there is no significant variation among male and female individuals.

**Table 2:** Length-length relationships parameters of bartail flathead, *Platycephalus indicus* from the Bay of Bengal, Bangladesh.

Sex	n	Equation	a	95% Cl of a	b	95% Cl of b	r^2^
Male	112	TL = a + bSL	2.959	2.605–3.313	1.075	1.064–1.086	0.996
		TL = a + bFL	2.518	2.142–2.894	1.033	1.022–1.044	0.996
		SL = a + bFL	−0.385	−0.645 to −0.126	0.960	0.952–0.967	0.998
Female	85	TL = a + bSL	3.582	3.190–3.974	1.054	1.043–1.065	0.997
		TL = a + bFL	2.598	2.107–3.090	1.029	1.015–1.042	0.996
		SL = a + bFL	−0.913	−1.286–0.540	0.975	0.965–0.985	0.997
Combined	197	TL = a + bSL	3.229	2.968–3.489	1.065	1.058–1.073	0.997
		TL = a + bFL	2.575	2.284–2.865	1.030	1.022–1.0390	0.996
		SL = a + bFL	−0.590	−0.802–0.378	0.966	0.960–0.972	0.998

Here, n: sample size, a: intercept, b: slope, r^2^: coefficient of determination.

### 3.4 Form factor (a_3.0_)

The determined form factor (a_3.0_) value of *P. indicus* was ranging from 0.0107 to 0.0112, and the maximum difference between the values was only 0.0005 (Table 1).

### 3.5 Condition factors

The K_F_ value ranged from 0.73 to 1.69 and the maximum K_F_ value was observed in the 33–36 cm TL groups in both males and females and the minimum K_F_ value was found in the 27–30 cm TL group (Table 3). According to Spearman rank correlation test, there were highly significant relationships between TL vs. K_F_ (r_s_ = −0.467 and p < 0.0001) and BW vs. K_F_ (r_s_ = −0.382 and p < 0.0001) (Table 4).

**Table 3:** Condition factors (K_F_ and K_A_) of bartail flathead, *Platycephalus indicus* in relation to size classes from the Bay of Bengal, Bangladesh.

TL	Total individuals	Fulton’s condition factor range	Mean ± SD	Allometric condition factor range	Mean ± SD
<21-23	14	0.91–1.26	1.10 ± 0.10	0.032–0.059	0.041 ± 0.011
24–27	31	0.81–1.69	1.09 ± 0.21	0.025–0.061	0.047 ± 0.014
27–30	9	0.73–1.54	1.05 ± 0.34	0.018–0.062	0.034 ± 0.023
30–33	6	1.17–1.49	1.37 ± 0.11	0.039–0.061	0.052 ± 0.012
33–36	6	1.35–1.66	1.48 ± 0.14	0.041–0.078	0.057 ± 0.009
36–39	9	1.14–1.50	1.29 ± 0.13	0.026–0.061	0.045 ± 0.005
39–42	54	0.98–1.34	1.14 ± 0.08	0.011–0.050	0.031 ± 0.016
42–45	32	0.98–1.37	1.08 ± 0.11	0.016–0.058	0.038 ± 0.012
45–48	19	1.00–1.21	1.10 ± 0.08	0.022–0.047	0.032 ± 0.001
>48	17	0.99–1.15	1.04 ± 0.04	0.021–0.049	0.035 ± 0.001

**Table 4:** Relationships of condition factors with total length and body weight of bartail flathead, *Platycephalus indicus* from the Bay of Bengal, Bangladesh.

Relationship	r_s_ value	r_s_ range	P value	Significance
TL vs. K_F_	−0.467	−0.834–0.047	<0.0001	∗∗∗
TL vs. K_A_	0.684	0.461–0.827	0.852	ns
TL vs. K_R_	0.073	0.058–0.092	0.035	∗
TL vs. W_R_	0.073	0.058–0.092	0.035	∗
BW vs. K_F_	−0.382	−0.972–0.278	0.0001	∗∗∗
BW vs. K_A_	0.728	0.502–0.981	0.529	ns
BW vs. K_R_	0.165	0.048–0.307	0.011	∗
BW vs. W_R_	0.165	0.048–0.307	0.011	∗

Here, TL: total length, BW: body weight, K_F_: Fulton′s condition factor, K_A_: allometric condition factor, K_R_: relative condition factor, W_R_: relative weight, r_S_: Spearman rank-correlation values, ns: not significant, ∗: significant, ∗∗∗: highly significant.

The K_A_ value ranged from 0.011 to 0.078 and the maximum K_A_ value was observed in the 33–36 cm TL groups in both males and females and the minimum K_A_ value was found in the 39–42 cm TL group (Table 3). According to Spearman rank correlation test, there were no significant relationships between TL vs. K_A_ (r_s_ = 0.684 and p = 0.852) and BW vs. K_A_ (r_s_ = 0.728 and p = 0.529) (Table 4).

The K_R_ value ranged from 0.76 to 1.73 and the maximum K_R_ value was observed in the 33–36 cm TL groups in both males and females and the minimum K_R_ value was found in the 27–30 cm TL group (Table 5). According to Spearman rank correlation test, there were significant relationships between TL vs. K_R_ (r_s_ = 0.073 and p = 0.035) and BW vs. K_R_ (r_s_ = 0.165 and p = 0.011) (Table 4).

**Table 5:** Condition factors (K_R_ and W_R_) of bartail flathead, *Platycephalus indicus* in relation to size classes from the Bay of Bengal, Bangladesh.

TL	Total individuals	Relative condition factor range	Mean ± SD	Relative weight range	Mean ± SD
<21–23	14	1.00–1.33	1.16 ± 0.11	100.09–132.81	115.76 ± 10.99
24–27	31	0.89–1.73	1.17 ± 0.21	88.85–173.04	116.60 ± 21.31
27–30	9	0.76–1.72	1.15 ± 0.40	76.28–172.13	115.27 ± 40.48
30–33	6	1.29–1.65	1.46 ± 0.11	128.99–165.02	145.67 ± 11.52
33–36	6	1.40–1.66	1.53 ± 0.11	139.88–165.59	153.07 ± 10.84
36–39	9	1.20–1.52	1.36 ± 0.10	120.20–152.43	136.33 ± 10.89
39–42	54	1.06–1.40	1.20 ± 0.08	106.14–139.91	120.38 ± 7.78
42–45	32	1.05–1.38	1.18 ± 0.09	104.51–137.63	118.60 ± 8.89
45–48	19	1.08–1.29	1.17 ± 0.08	108.11–128.87	117.21 ± 8.57
>48	17	1.09–1.26	1.16 ± 0.03	109.51–126.13	116.49 ± 3.27

The W_R_ value ranged from 76.28 to 173.04 and the maximum W_R_ value was observed in the 33–36 cm TL groups in both males and females and the minimum W_R_ value was found in the 27–30 cm TL group (Table 5). According to Spearman rank correlation test, there were significant relationships between TL vs. W_R_ (r_s_ = 0.073 and p = 0.035) and BW vs. W_R_ (r_s_ = 0.165 and p = 0.011) (Table 4).

### 3.6 Size at first sexual maturity (L_m_)

The L_m_ for combined sex of *P. indicus* was calculated in the present study as 30.2 cm total length in the Bay of Bengal, Bangladesh.

### 3.7 Natural mortality (M_w_)

**Figure 4:**
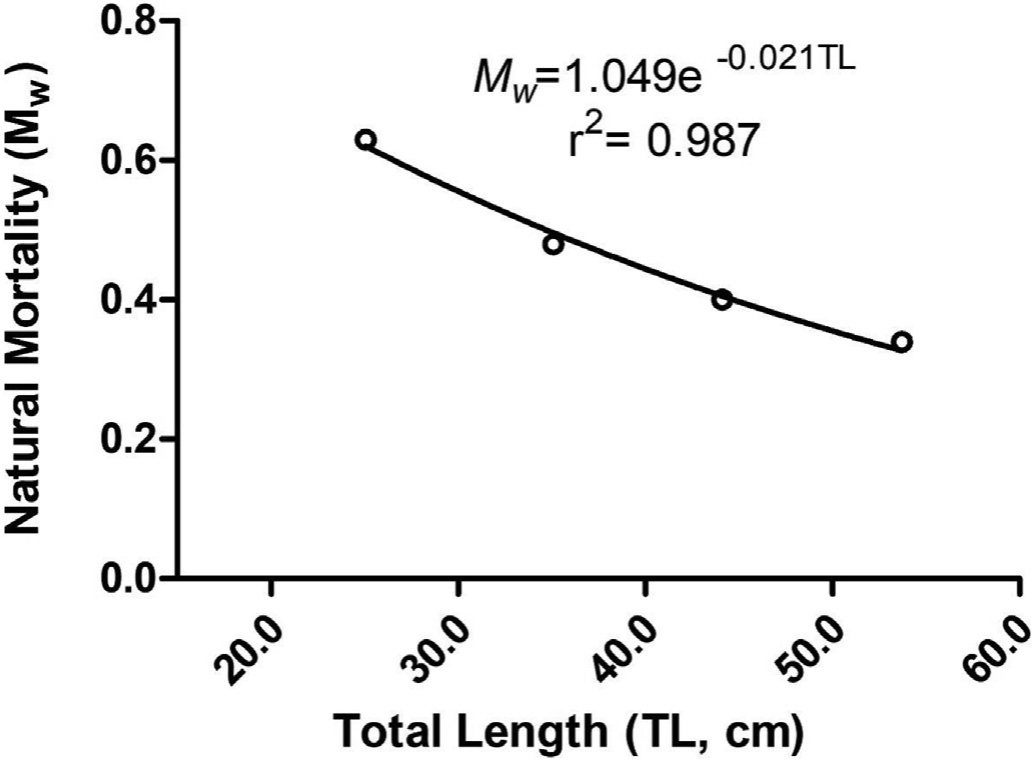
Natural mortality (M_w_) of *P. indicus* from the Bay of Bengal, Bangladesh.

In the present study, the mean natural mortality (M_w_) for the *P. indicus* population was calculated as 0.46 year^−1^ in the Bay of Bengal, Bangladesh and it is shown in Figure 4.

## 4 Discussion

The sex ratio is important in population demographics, and it may be influenced by anthropogenic pressures like selective fishing (Mredul et al., 2021). Our study revealed that gender-biased fishing was not conducted during the study period, however dominant male population was reported due to high fishing pressure. The sex ratio can fluctuate from population to population, or even within the same population at various times, depending on a variety of factors such as population adaptability, reproductive function, food sources, and characteristics of the environment (Vandeputte et al., 2012). Female reproductive success is typically linked to resource availability and environmental conditions, rather than the number of mating partners, as it is for males. As a result, the successful reproductive performance of males is restricted by the availability of females, whilst the efficiency of female reproduction is unaffected by access to males, resulting in an imbalance in the population’s number of each sex (Forsgren et al., 2008).

Estimates of the parameter b (2.978 and 3.297) obtained from linear regressions of the relationship between log weight and total length demonstrate that the patterns of growth of females, males, and the combination of females and males of *P. indicus* from the Bay of Bengal all exhibit positive allometry. A similar outcome (b = 3.2299) was also reported by Mohammadikia et al. (2014) for the combined data for females and males of this species from the Persian Gulf. Isometric growth means that the body expands in all aspects at the same rate, whereas positive allometry means that the body gets fatter as it grows longer, and negative allometry means that the body gets slimmer (Jobling, 2002). If b is greater than 3, the pattern of growth exhibits positive allometry; if b is less than 3, the pattern of growth exhibits negative allometry; and if b is equal to 3, the growth pattern is isometric (Bagenal and Tesch, 1978). Although values of the allometric coefficient (b) of LWRs can vary from 2.0 to 4.0, they normally lie between 2.5 and 3.5 (Carlander, 1969; Froese, 2006). Environmental variables, gonad development stages, sex, stomach fullness, functional health state, season, population, and variation among species all influence the parameters of L-W interactions in fish (Nieto-Navarro et al., 2010).

The lack of *P. indicus* with total lengths less than 21 cm in samples collected for the current study is attributed to the selectivity of the fishing gear (Hossain et al., 2012). In addition, the greatest length of *P. indicus* inside the sampling sites was 55.9 cm TL, somewhat less than the largest documented value of 58 cm TL (Mohammadikia et al., 2014). Hashemi et al. (2012) reported the minimum size group of this species was 5.7 cm. In principle, maximum size is recognized as a valuable tool for fisheries resource utilization and conservation, and it is essential for determining asymptotic growth and length coefficient (Khatun et al., 2019; Saha et al., 2021). LLR involves the relation between TL and the other three-length parameters such as Fork length (FL), Standard length (SL) & Body width (BWth); the equation is Y = bx + a, and the regression equation is shown in Figure 3. At a 95% level of significance, all correlation coefficient (r^2^) findings are highly correlated. The slope (b) of LLRs is close to 1.0, indicating that the three-length parameters expand simultaneously as the total length (TL). The relationship between total length, standard length, and fork length with body width is closely connected, which reflects a homogenous growth pattern. These variables are critical in determining the maximum permissible catch and mesh size to utilize. The assessed form factor (a_3.0_) score in this investigation ranged from 0.0107 to 0.0112. This number is commonly used to determine whether an individual’s body shape in a certain fish population or species differs from that of other species (Froese, 2006). However, no form factor reference value has been identified in the literature for this species. So, the current form factor results will serve as a valuable baseline for future comparisons.

Several condition factors (K_F_, K_A_, K_R_, and W_R_) were calculated in the present study to assess the well-being and health status of *P. indicus* from the Bay of Bengal, Bangladesh though most of the investigations address only a single condition factor. Condition factors based on the LWRs are a good indicator of general fish condition and food reserves (Offem et al., 2007). The Spearman correlation values (r_s_) showed a positive value between total length (TL) and condition factors (K_A_, K_R_, W_R_) as also did so between weight (BW) and condition factors (K_A_, K_R_, W_R_) (Table 4). But within those four condition factors, only Fulton’s condition factor (K_F_) showed different correlation values between TL vs. K_F_ (r_s_ = −0.467) and BW vs. K_F_ (r_s_ = −0.382). Fulton’s condition factor (K_F_) may thus be the best method for evaluating the condition of this population in Bangladesh’s Bay of Bengal as well as the surrounding environment. Fluctuation in condition factors indicates the gonad weight increase or decrease during the reproduction cycle. According to Ali et al. (2014) many elements, including sex, seasonality, environmental conditions, stress, gonadal maturation, and the availability of prey items, govern the condition factor overall. Moreover, food supply, floods, heat, temperature variations, pH, water pollution, and other factors might cause a rise or reduction in the condition factor (Osman et al., 2019).

An important predictor of the minimum allowable catch size is the size at first sexual maturity, or L_m_, which is particularly significant for fish stock assessment (Hossain et al., 2019). According to this study, the size at first sexual maturity for *P. indicus* in Bangladesh’s Bay of Bengal was 30.2 cm. This study is the first to attempt to determine the size at first sexual maturity for *P. indicus* in both Bay of Bengal and global waters. Moreover, the natural mortality of the *P. indicus* in the Bay of Bengal, Bangladesh for the entire population was expected to be 0.46 year^−1^, allowing it the first worldwide evaluation. However, it is the fundamental data for future fisheries biology studies.

## 5 Conclusion

In closing, our study is the first one to give specific information on *P. indicus* biometric traits in Bangladesh’s large coastal habitat. The length-length relationships have a strong correlation. Almost all LWRs had positive allometric fish growth, which might be attributed to environmental conditions. The results of this study provide valuable benchmark data for coastal and marine fisheries management of *P. indicus* in the Bay of Bengal against which to assess future deleterious changes in sex ratio or conditions arising from anthropogenic pressures or environmental change. For example, knowing the species' growth rate will aid in the protection of this valuable fishery resource and the implementation of appropriate fishing laws to lessen fishing pressure. However, in order to effectively preserve and conserve this species, more detailed research on recruitment pattern, reproductive physiology, and gonad development is required.

## Declarations

### Author contribution statement

Md. Rahamat Ullah: Conceptualization; Methodology, Investigation, Data collection and analysis; Fund acquisition; Writing – original draft, Finalising the manuscript.

Md Mahamudul Hasan Mredul: Data analysis; Validation; Writing – review & editing; Finalising the manuscript.

### Funding statement

Md. Rahamat, Ullah was supported by National Science and Technology Fellowship (NST-2019-20).

### Data availability statement

Data will be made available on request.

### Declaration of interests statement

The authors declare no conflict of interest.

### Additional information

No additional information is available for this paper.

## References

[bib1] Ali S., Barat A., Kumar P., Sati J., Kumar R., Haldar R.S. (2014). Study of length-weight relationship and condition factor of the Golden mahseer, *Tor putitora* from Himalayan rivers of India. J. Environ. Biol..

[bib2] Andrade H.A., Camos R.O. (2002). Allometry coefficient variations of the length-weight relationship skipjack tuna (*Katsuwonus pelamis*) caught in the southwest South Atlantic. Fish. Res..

[bib3] Badcock J., Merrett N.R. (1976). Midwater fishes in the eastern North Atlantic. I. Vertical distribution and associated biology in 30°N, 23°W, with developmental notes on certain myctophids. Prog. Oceanogr..

[bib4] Bagenal T.B., Tesch F.W., Bagenal T. (1978). Methods of Assessment of Fish Production in Fresh Waters.

[bib5] Batubara A.S., Muchlisin Z.A., Efizon D., Elvyra R., Irham M. (2019). Length-weight relationships and condition factors of the Naleh fish, *Barbonymus gonionotus* (Pisces, Cyprinidae) harvested from Nagan Raya waters, Indonesia. Vestn. Zool..

[bib6] Binohlan C., Froese R. (2009). Empirical equations for estimating maximum length from length at first maturity. J. Appl. Ichthyol..

[bib7] Bogard J.R., Thilsted S.H., Marks G.C., Wahab M.A., Hossain M.A.R., Jakobsen J., Stangoulis J. (2015). Nutrient composition of important fish species in Bangladesh and potential contribution to recommended nutrient intakes. J. Food Compos. Anal..

[bib8] Bolarinwa J.B. (2017). Length-weight relationships and condition factors of *Cynoglossus Cynoglossus* and *Caranx hippos* in Epe Lagoon, Nigeria. Int. J. Res. Agric. For..

[bib9] Carlander K.D. (1969).

[bib10] Chen Z., Song N., Zou J., Qin Y., Ma L., Gao T. (2020). Identification of species in genus *Platycephalus* from seas of China. J. Ocean Univ. China.

[bib11] Cheng Q., Zheng B. (1987). Systematic Synopsis of Chinese Fishes.

[bib12] Clarke T.A. (1983). Sex ratios and sexual differences in size among mesopelagic fishes from the central Pacific Ocean. Mar. Biol..

[bib13] Clutton-Brock T. (2007). Sexual selection in males and females. Science.

[bib14] Forsgren E., Reynolds J.D., Berglund A., Mooi R.D. (2008). Behavioural ecology of reproduction in fish. Handb. Fish Biol. Fish..

[bib15] Froese R. (2006). Cube law condition factor and weight-length relationships: history, meta-analysis and recommendations. J. Appl. Ichthyol..

[bib16] Froese R., Pauly D., Day F. (2007).

[bib17] Fulton T.W. (1904).

[bib18] Hashemi S.A., Taghavimotlagh S.A. (2013). Population parameters and length-weight relationship of bartail flathead (*Platycephalus indicus* Linnaeus, 1758) in Northwest Persian Gulf (Khuzestan coastal waters, Iran). World J. Fish Mar. Sci..

[bib19] Hashemi S.A., Taghavimotlagh S.A., Eskandary G. (2012). Some biological aspect of bartail flathead (*Platycephalus indicus*, Linnaeus, 1758) in Northwest of Persian Gulf (Khuzestan coastal waters, Iran). World J. Fish Mar. Sci..

[bib20] Hossain M.Y., Rahman M.M., Miranda R., Leunda P.M., Oscoz J., Jewel M.A.S., Naif A., Ohtomi J. (2012). Size at first sexual maturity, fecundity, length-weight and length-length relationships of *Puntius sophore* (Cyprinidae) in Bangladeshi waters. J. Appl. Ichthyol..

[bib21] Hossain M.A., Hossain M.Y., Hossen M.A., Rahman M.A., Islam M.A., Khatun D., Nawer F., Ohtomi J. (2019). Temporal variations of sex ratio and growth pattern of critically endangered catfish *Clupisoma garua* from the Ganges River of north-western Bangladesh. Indian J. Geo-Mar. Sci..

[bib22] Huang L.M., Wang J., Li J., Zhang Y.Z., Shen S.C. (2018). Length-weight relationships of 15 fish species in the Amoy Bay, East China sea. J. Appl. Ichthyol..

[bib23] Imamura H. (2015). Taxonomic revision of the flathead fish genus *Platycephalus* Bloch, 1785 (Teleostei: Platycephalidae) from Australia, with description of a new species. Zootaxa.

[bib24] Jobling M. (2002). Environmental factors and rates of development and growth. Handb. Fish Biol. Fish..

[bib25] Kassem S., Ibrahim G., Mousa M., Ahmed M. (2021). Length-weight relationship and condition factor of the bartail flathead (*Platycephalus indicus*) in Bardawil lagoon, north Sinai, Egypt. Sinai J. Appl. Sci..

[bib26] Khatun D., Hossain M.Y., Rahman M.A., Islam M.A., Rahman O., Azad M.A.K., Sarmin M.S., Parvin M.F., Haque A.T.U., Mawa Z., Hossain M.A. (2019). Life-history traits of the climbing perch *Anabas testudineus* (Bloch, 1792) in a wetland ecosystem. Jordan J. Biol. Sci..

[bib27] Kuiter R.H., Tonozuka T. (2001).

[bib28] Le Cren E.D. (1951). The length-weight relationship and seasonal cycle in gonad weight and condition in the perch (*Perca fluviatilis*). J. Anim. Ecol..

[bib29] Linnaeus C. (1758). Systema Naturae, Ed. X. (Systema naturae per regna tria naturae, secundum classes, ordines, genera, species, cum characteribus, differentiis, synonymis, locis. Tomus I. Editio decima, reformata.) Holmiae. Syst. Nat..

[bib30] Mohammadikia D., Kamrani E., Reza-Taherizadeh M., Soleymani A., Farokhi E., Momeni M. (2014). Age and growth of flathead, *Platycephalus indicus* from the Persian Gulf (Bandar Abbas, Iran). J. Mar. Biol. Assoc. U. K..

[bib31] Mousavi-Sabet H., Heidari A., Fekrandish H. (2015). Population structure, length-weight and length-length relationships of six populations of the Bartail Flathead *Platycephalus indicus* (Scorpaeniformes: Platycephalidae) along the Persian Gulf coastal waters. J. Threat. Taxa.

[bib32] Mredul M.M.H., Alam M.R., Akkas A.B., Sharmin S., Pattadar S.N., Ali M.L. (2021). Some reproductive and biometric features of the endangered Gangetic Leaf Fish, *Nandus nandus* (Hamilton, 1822): implication to the baor fisheries management in Bangladesh. Aquac. Fish..

[bib33] Muchlisin Z.A., Fransiska V., Muhammadar A.A., Fauzi M., Batubara A.S. (2017). Length-weight relationships and condition factors of the three dominant species of marine fishes caught by traditional beach trawl in Ulelhee Bay Banda Aceh City, Indonesia. Croat. J. Fish..

[bib34] Muchlisin Z.A., Siti-Azizah M.N. (2009). Diversity and distribution of freshwater fishes in Aceh waters, northern Sumatera, Indonesia. Int. J. Zool. Res..

[bib35] Nash R.D., Valencia A.H., Geffen A.J. (2006). The origin of Fulton’s condition factor-setting the record straight. Fisheries.

[bib36] Nelson J.S. (2006).

[bib37] Nieto-Navarro J.T., Zetina- Rejon M., Arreguin- Sanchez F., Arcos- Huitron N.E., Pena- Messina E. (2010). Length-weight relationship of demersal fish from the Eastern coast of the mouth of the Gulf of California. J. Fish. Aquat. Sci..

[bib38] Offem B.O., Akegbejo-Samsons Y., Omoniyi I.T. (2007). Biological assessment of *Oreochromis niloticus* (Pisces: Cichlidae: Linne, 1958) in a tropical floodplain river. Afr. J. Biotechnol..

[bib39] Ogunola O.S., Onada O.A. (2017). Preliminary investigation of length-weight relationships and condition factors of two commercially important fish species (Mullet, *Mugil cephalus* (Linnaeus 1758) and Sardine, *Sardinella maderensis*. (Lowe 1838)) in Okrika creeks (Niger-Delta) of Nigeria. Reg. Stud. Mar. Sci..

[bib40] Oluwatoyin A., Akintade A., Edwin C., Victor K. (2013). A study of length weight relationship and condition factor of west African blue crab (*Callinectes pallidus*) from Ojo Creek, Lagos, Nigeria. Am. J. Res. Commun..

[bib41] Oscoz J., Campos F., Escala M.C. (2005). Weight–length relationships of some fish species of the Iberian Peninsula. J. Appl. Ichthyol..

[bib42] Osman H.M., Saber M.A., El Ganainy A.A. (2019). Population structure of the striped piggy *Pomadasys stridens* in the Gulf of Suez. Egypt. J. Aquat. Res..

[bib43] Parvin M.F., Hossain M.Y., Rahman M.A., Khatun D., Sarmin M.S., Rahman O., Islam M.A., Azad M.A.K., Samad M.A., Sabbir W., Kamruzzanan S., Hosneara U., Ul-Hassan H. (2021). Growth, maturity, condition, size at sexual maturity and mortality of the Banded gourami *Trichogaster fasciata* from the Ganges River, Northwestern Bangladesh. Egypt. J. Aquat. Biol. Fish..

[bib44] Peterson I., Wroblewski J.S. (1984). Mortality rates of Fishes in the pelagic ecosystem. Can. J. Fish. Aquat. Sci..

[bib45] R Core Team (2020). R: A Language and Environment for Statistical Computing. http://www.R-project.org.

[bib46] Rahman M.A., Hossain M.A., Ullah M.R., Iqbal M.M. (2020). Reproductive biology of Gagora catfish (*Arius gagora*) at Meghna river system, Kushiara river, Bangladesh. Int. J. Aquat. Biol..

[bib47] Riede K. (2004).

[bib48] Saha N., Rakib M.H., Mredul M.M.H., Rahman M.A., Ahamed F. (2021). Life history traits of the Gangetic scissortail rasbora, *Rasbora rasbora* (Hamilton, 1822) in the Payra River, southern Bangladesh. Jordan J. Biol. Sci..

[bib49] Saha N., Ullah M.R., Islam M.S., Hossain M.B. (2019). Morphometric relationships between length-weight and length-length and condition factor of four small indigenous fishes from the Payra River, southern Bangladesh. Arch. Agric. Environ. Sci..

[bib50] Talwar P.K., Jhingran A.G. (1991).

[bib51] Tesch F.W., Ricker W.E. (1971). Methods for Assessment of Fish Production in Fresh Waters.

[bib52] Vandeputte M., Quillet E., Chatain B. (2012). Are sex ratios in wild European sea bass (*Dicentrarchus labrax*) populations biased?. Aquat. Living Resour..

